# Age estimates for an adaptive lake fish radiation, its mitochondrial introgression, and an unexpected sister group: Sailfin silversides of the Malili Lakes system in Sulawesi

**DOI:** 10.1186/1471-2148-14-94

**Published:** 2014-05-03

**Authors:** Björn Stelbrink, Isabella Stöger, Renny K Hadiaty, Ulrich K Schliewen, Fabian Herder

**Affiliations:** 1 Museum für Naturkunde Leibniz-Institute für Evolutions- und Biodiversitätsforschung an der Humboldt, Universität zu Berlin, Invalidenstr. 43, D-10115 Berlin, Germany; 2 Department of Ichthyology, Bavarian State Collection of Zoology (ZSM), Münchhausenstr. 21, D-81247 München, Germany; 3 Museum Zoologicum Bogoriense, Ichthyology Laboratory, Division of Zoology, Research Center for Biology, Indonesian Institute of Sciences (LIPI), Jl, Raya Bogor Km 46, 16911 Cibinong, Indonesia; 4 Zoologisches Forschungsmuseum Alexander Koenig, Sektion Ichthyologie, Adenauerallee 160, D-53113 Bonn, Germany

**Keywords:** Molecular clock, Mitochondrial DNA, Southeast Asia, Sulawesi, Biogeography, Adaptive radiation, Introgressive hybridization

## Abstract

**Background:**

The Malili Lakes system in central Sulawesi (Indonesia) is a hotspot of freshwater biodiversity in the Wallacea, characterized by endemic species flocks like the sailfin silversides (Teleostei: Atherinomorpha: Telmatherinidae) radiation. Phylogenetic reconstructions of these freshwater fishes have previously revealed two Lake Matano *Telmatherina* lineages (sharpfins and roundfins) forming an ancient monophyletic group, which is however masked by introgressive hybridization of sharpfins with riverine populations. The present study uses mitochondrial data, newly included taxa, and different external calibration points, to estimate the age of speciation and hybridization processes, and to test for phylogeographic relationships between *Kalyptatherina* from ancient islands off New Guinea, *Marosatherina* from SW Sulawesi, and the Malili Lakes flock.

**Results:**

Contrary to previous expectations, *Kalyptatherina* is the closest relative to the Malili Lakes Telmatherinidae, and *Marosatherina* is the sister to this clade. Palaeogeographic reconstructions of Sulawesi suggest that the closer relationship of the Malili Lakes radiation to *Kalyptatherina* might be explained by a 'terrane-rafting’ scenario, while proto-*Marosatherina* might have colonized Sulawesi by marine dispersal. The most plausible analysis conducted here implies an age of c. 1.9 My for the onset of divergence between the two major clades endemic to Lake Matano. Diversification within both lineages is apparently considerably more recent (c. 1.0 My); stream haplotypes present in the sharpfins are of even more recent origin (c. 0.4 My).

**Conclusions:**

Sulawesi’s Telmatherinidae have most likely originated in the Sahul Shelf area, have possibly reached the island by both, marine dispersal and island/terrane-rafting, and have colonized the Malili Lakes system from rivers. Estimates for the split between the epibenthic sharpfins and the predominantly pelagic to benthopelagic roundfins in Lake Matano widely coincide with geological age estimates of this rift lake. Diversification within both clades clearly predates hybridization events with stream populations. For Lake Matano, these results support a scenario of initial benthic-pelagic divergence after colonization of the lake by riverine populations, followed by rapid radiation within both clades within the last 1 My. Secondary hybridization of stream populations with the sharpfins occurred more recently, and has thus most likely not contributed to the initial divergence of this benthic species flock.

## Background

Adaptive radiations of organisms restricted to habitat islands are among the prime model systems for investigating speciation processes in nature [1-4]. Estimations of rates of radiation, as well as the reconstruction of past geographic scenarios of divergence, require the incorporation of a temporal axis into phylogenetic reconstructions [5-7]. Likewise, the dating of phylogenetic splits may contribute to the understanding of other processes potentially contributing to evolutionary divergence (and adaptive radiation), such as introgressive hybridization [8,9].

The ancient lakes of Sulawesi’s central highlands are a hotspot of aquatic diversity, strongly dominated by endemic species flocks, including radiations of freshwater fishes [10]. The Malili Lakes system (Matano, Mahalona, and Towuti) – a hydrological chain of three main lakes interconnected by rivers – is the main habitat of the sailfin silversides radiation (Atherinomorpha: Telmatherinidae). About 30 morphospecies of these sexually dimorphic fishes that typically possess bright male colourations are distinguished in the Malili Lakes drainage and a few surrounding rivers [11]. Sailfin silversides show conspicuous colour polymorphisms [12,13], and there are clear indications that ecological speciation has shaped their adaptive radiation [14-16]. Phylogenetic analyses suggest that the two lineages of *Telmatherina* radiating in the hydrological head of the lakes system, ancient graben-lake Matano, form an ancient monophyletic group that was later compromised by introgressive hybridization from stream populations [17]. This introgression has affected only the “sharpfins”, a lineage of predominantly epibenthic sailfin silversides, whereas their rather pelagic sister group, the “roundfins”, show no indications of genetic exchange with stream populations ( [17,18]; see [19] for discussion).

Morphological data support a clade composed of *Marosatherina ladigesi* from south-west Sulawesi, and *Kalyptatherina helodes* from the islands Batanta and Misool off the Vogelkop peninsula (Birds’ Head, New Guinea), as sister group of the Telmatherinidae in and close by the Malili Lakes [20]. However, only *Marosatherina* has been considered as an outgroup to the lakes radiation in genetic studies so far [11,21]. Likewise, Telmatherinidae are represented only by *Marosatherina*, a species available worldwide by the aquarium trade, in most phylogenetic studies targeting relationships within the Atherinomorpha (e.g., [22-24]).

Here, we use mitochondrial data to (i) assess the relationship of *Kalyptatherina* relative to *Marosatherina* and the Malili Lakes Telmatherinidae, (ii) estimate the age of the sailfin silverside radiation of the Malili Lakes, and (iii) provide an estimation of the age of the mitochondrial introgression present in Lake Matano’s sharpfin *Telmatherina*. For this, we combine sequence data of the Telmatherinidae with data of representatives of the Melanotaeniidae, the closely related rainbowfishes from Australia and New Guinea [22-25], and estimate divergence times using both indirect and geological calibration points, and substitution rates suggested by [24] as telmatherinid fossils are missing.

## Methods

### DNA extraction, amplification and sequencing

DNA from 99 specimens, representing 74 taxa or populations (Additional file 1), was extracted using the QIAGEN DNeasy® Blood & Tissue Kit following the manufacturers’ instructions. Two mitochondrial loci were amplified by using the Sigma Taq-Polymerase system: partial NADH dehydrogenase subunit 2 (ND2, 830 bp length) and a combined fragment comprising partial 12S rRNA, tRNA-Val, and partial 16S rRNA (“12S-16S fragment”; ca. 1,275 bp length). All PCR reactions were conducted using the same conditions: 6 min at 94°C (initial denaturation); 45 cycles: 1 min at 94°C (denaturation); 1 min at 45°C (annealing); 1.5 min at 72°C (elongation). Two sets of primer pairs (ND2) and three different primers (12S-16S fragment) were used for both amplification and sequencing (Table 1). The 12S-16S fragment could not be amplified for all *Kalyptatherina helodes* specimens; the same applies to the ND2 fragment for *Melanotaenia lacustris* (AP00419) and *Melanotaenia boesemani*. Missing data was replaced by Ns for these specimens. PCR products were purified using the enzymatic digestion system USB ExoSAP-It. Sequencing was carried out by the Sequencing Service of the Ludwig-Maximilians-Universität Munich, Department of Genetics using a ABI 3730 48 capillary sequencer. All sequences are deposited in GenBank at the NCBI [ND2, GenBank:KJ667866-KJ667963; 12S-16S, GenBank:KJ667771-KJ667865].

**Table 1:** **Primers used in the present study (seeMethodsfor PCR conditions)**

**Primer name**	**Sequence**	**Source**
ND2Met	5′-CAT ACC CCA AAC ATG TTG GT-3′	[26]
ND2Trp	5′-GTS GST TTT CAC TCC CGC TTA-3′	[26]
ND2Gln	5′-CTA CCT GAA GAG ATC AAA AC-3′	[26]
ND2Asn	5′-CGC GTT TAG CTG TTA ACT AA-3′	[26]
12SF1	5′-TGA AGG AGG ATT TAG CAG TAA G-3′	[27]
12SF2	5′-TCT CTG TGG CAA AAG AGT-3′	[27]
16SR1	5′-AAG TGA TTG CGC TAC CTT CGC AC-3′	[27]

### Phylogenetic analyses and estimation of divergence times

Single gene sequences were aligned using MAFFT ( [28]; default settings; http://www.ebi.ac.uk/Tools/msa/mafft) and corrected by eye; ambiguous sites at the 5′ end of the 12S-16S fragment were removed manually. Both single gene alignments were concatenated using SequenceMatrix v. 1.7.8. [29] resulting in a total alignment of 2,102 bp. The GTR + G substitution model was used for maximum likelihood (ML) analyses using RAxML BlackBox ( [30]; partition model; 100 bootstrap replicates) and Bayesian inference (BI) using MrBayes v. 3.1.2 ( [31]; partition model; ngen = 10,000,000; nchains = 4; samplefreq = 500; burnin = 10,001). *Iso rhotophilus* was used as outgroup in all analyses as suggested in [32]; phylograms were visualized and re-rooted in FigTree v. 1.4 (available at: http://tree.bio.ed.ac.uk/software/figtree).

**Figure 1:**
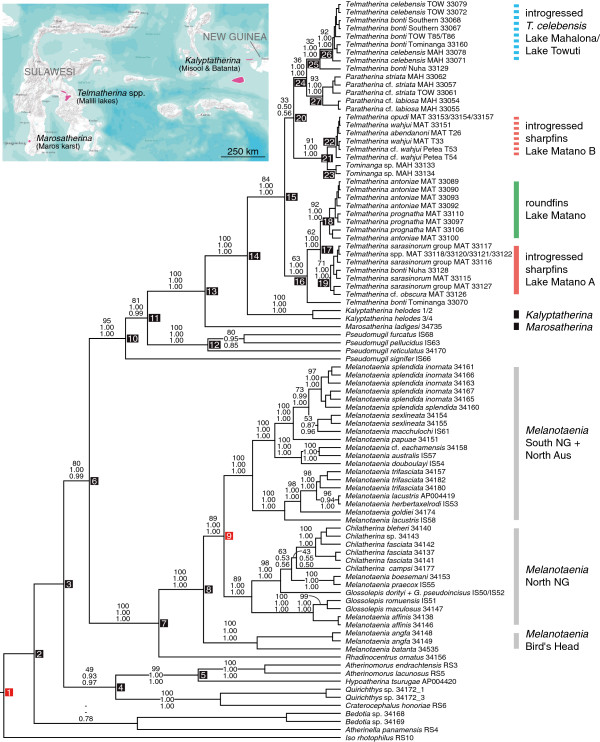
**BEAST MCC tree showing general melanotaeniid and telmatherinid relationships for the concatenated dataset.** Topology derived from analysis [D]. Numbers on branches denote RAxML bootstrap values, MrBayes posterior probabilities and BEAST posterior probabilities (from top to bottom); numbers on nodes correspond to node numbers given in Table 2, i.e., divergence time estimates across all four analyses performed. Nodes 1 and 9 were used as indirect and geological calibration points, respectively (see Methods for details). Abbreviations used for sailfin silversides: MAH = Lake Mahalona; MAT = Lake Matano; TOW = Lake Towuti. See text and Figure 3 for details on the Lake Matano telmatherinid radiation. Map shows current distribution of *Kalyptatherina*, *Marosatherina*, and the Malili Lakes telmatherinds.

Single-gene datasets were reduced to unique haplotypes using DAMBE v. 5.1.1 [28]. Substitution models were selected using jModelTest ( [33]; AIC and BIC selected GTR + G for both partitions). Divergence times were estimated in BEAST v. 1.7.4 [34] on the CIPRES Science Gateway web portal [35]. Each analysis was run twice using the following settings: separate partitions used; ingroup monophyly enforced; uncorrelated lognormal relaxed-clock model; ngen = 40,000,000; samplefreq = 2,000; burnin = 10,001; Yule model; calibration points with normal distribution; the RAxML tolopolgy was used as starting tree for all analyses. Four different analyses were: [A] indirect calibration of the root height using the estimated divergence time of the split of *Iso*–*Melanotaenia* obtained from [36] (93–113 Mya): Root height was set to 106.0 +/-10.0 My; [B] indirect calibration of the melanotaeniid northern–southern split inferred from a recent molecular clock analysis by [24] (23.8-30.8 My): Node 9 (see Figure 1), representing the split of northern and southern clades of New Guinean melanotaeniids was set to 27.3 +/-3.5 My; [C] a separate run was performed using a substitution rate of 1%/My for mtDNA suggested and used by [24]; [D] geological calibration of the Central Highlands formation in New Guinea (10–14 Mya; [37]) putatively resulting in the geographical separation of the two major melanotaeniid clades (“northern” and “southern”; see also [24]): Node 9 (see Figure 1), representing the split of northern and southern clades of New Guinean melanotaeniids and is probably related to the abovementioned orogeny was set to 12 +/-2.0 My. See Table 2 for results among datasets. Log files of replicates were visualized in Tracer v. 1.5 [38] for congruency and combined in LogCombiner v. 1.7.4 (BEAST package; 50% burnin). Log files were visualized in Tracer v. 1.5; ESS values for each parameter never dropped below 200 except for analysis [C] (low values for some parameters such as 'prior’ and 'posterior’ , however 'likelihood’ is >1,000). Tree files were combined in LogCombiner v. 1.7.4 (50% burnin) and summarized in TreeAnnotator v. 1.7.4 (BEAST package; no burnin; MCC trees = maximum clade credibility trees).

**Table 2:** Divergence time estimates for selected nodes

	**Mean age (lower and upper 95% HPD) [My]**			
**Analysis**	**[A]**	**[B]**	**[C]**	**[D]**
**Node**				
1	**103.09 (82.70, 122.15)**	70.36 (39.70, 106.48)	33.15 (23.43, 43.20)	31.31 (16.40, 48.90)
2	93.46 (69.89, 116.89)	64.19 (36.83, 95.98)	30.46 (22.53, 39.22)	28.65 (14.92, 43.70)
3	83.62 (59.92, 107.04)	57.69 (33.54, 85.19)	27.47 (20.40, 34.95)	26.03 (13.56, 39.08)
4	67.20 (40.38, 93.53)	46.10 (22.62, 70.57)	21.88 (13.85, 31.38)	20.40 (9.07, 32.21)
5	41.45 (18.04, 65.92)	22.64 (8.27, 39.89)	10.44 (3.89, 17.11)	9.95 (3.14, 18.18)
6	75.85 (53.25, 98.86)	52.00 (29.69, 76.73)	24.94 (18.31, 31.63)	23.56 (12.17, 35.75)
7	54.59 (35.08, 75.21)	39.49 (24.69, 56.03)	18.74 (13.30, 24.86)	17.41 (9.49, 26.50)
8	40.78 (24.86, 58.33)	30.34 (20.55, 40.64)	13.09 (9.22, 13.73) | 32.7 (28.4, 37.4)	12.74 (7.54, 18.43)
9	34.50 (20.65, 49.72)	**25.85 (18.83, 32.88)**	11.04 (7.69, 14.78) | 27.0 (23.8, 30.8)	**10.87 (6.70, 14.98)**
10	64.76 (43.47, 86.22)	43.85 (25.27, 65.90)	21.35 (15.20, 27.93)	20.19 (10.38, 30.97)
11	57.80 (37.80, 78.33)	38.95 (22.54, 59.33)	19.10 (13.38, 25.44)	18.11 (9.05, 27.98)
12	37.88 (18.74, 56.83)	25.00 (11.00, 39.86)	13.15 (6.95, 19.24)	12.30 (4.90, 20.65)
13	42.86 (25.60, 62.34)	27.17 (14.37, 42.94)	13.60 (8.89, 18.66)	12.93 (6.18, 20.36)
14	28.90 (15.01, 43.48)	19.15 (9.56, 30.43)	9.11 (5.50, 13.06)	8.45 (3.79, 13.45)
15	17.43 (8.21, 27.65)	11.99 (5.78, 19.06)	5.35 (3.22, 8.12)	5.18 (2.15, 8.53)
16	9.59 (3.52, 17.08)	7.13 (3.21, 12.20)	3.12 (1.47, 4.91)	3.00 (1.01, 5.28)
17	6.43 (2.40, 11.60)	4.45 (1.95, 7.62)	1.98 (0.90, 3.20)	1.86 (0.63, 3.27)
18	3.66 (1.18, 7.03)	2.45 (0.81, 4.31)	1.04 (0.39, 1.82)	1.01 (0.31, 1.92)
19	3.05 (0.90, 5.81)	2.24 (0.73, 4.15)	0.99 (0.36, 1.78)	0.93 (0.27, 1.80)
20	13.94 (6.35, 22.44)	10.15 (4.84, 16.54)	4.40 (2.32, 6.70)	4.32 (1.72, 7.17)
21	3.60 (0.73, 7.77)	2.92 (0.64, 6.29)	1.27 (0.33, 2.68)	1.22 (0.25, 2.68)
22	1.21 (0.22, 2.61)	0.92 (0.17, 1.98)	0.40 (0.09, 0.83)	0.37 (0.07, 0.83)
23	1.20 (0.08, 2.97)	0.96 (0.08, 2.44)	0.41 (0.04, 1.00)	0.38 (0.02, 0.95)
24	10.23 (4.58, 17.24)	7.24 (3.09, 11.98)	3.18 (1.54, 5.07)	3.03 (1.19, 5.33)
25	6.14 (2.08, 11.43)	4.39 (1.44, 8.03)	1.92 (0.73, 3.42)	1.82 (0.48, 3.45)
26	2.36 (0.64, 4.63)	1.68 (0.50, 3.26)	0.73 (0.23, 1.34)	0.70 (0.18, 1.37)
27	5.05 (1.54, 9.36)	3.84 (1.09, 7.02)	1.67 (0.61, 3.02)	1.61 (0.48, 3.07)
Resulting rate [% / My]	0.33 (ucld.mean)	0.47 (ucld.mean)	**1.00 (ucld.mean)**	1.14 (ucld.mean)

Bold-marked cells denote nodes used for prior calibration; [A]: root height = 106.0 +/-10.0 My; [B]: 27.3 +/-3.5 My; [C]: relaxed clock = 1%/My – node 8 and 9 show striking differences between the present analysis (first column) and the node ages inferred by [24] (second column); [D]: 12.0 +/-2.0 My. See Methods and Figure 1 for details.

## Results

### Phylogenetic relationships and age estimates

**Figure 2:**
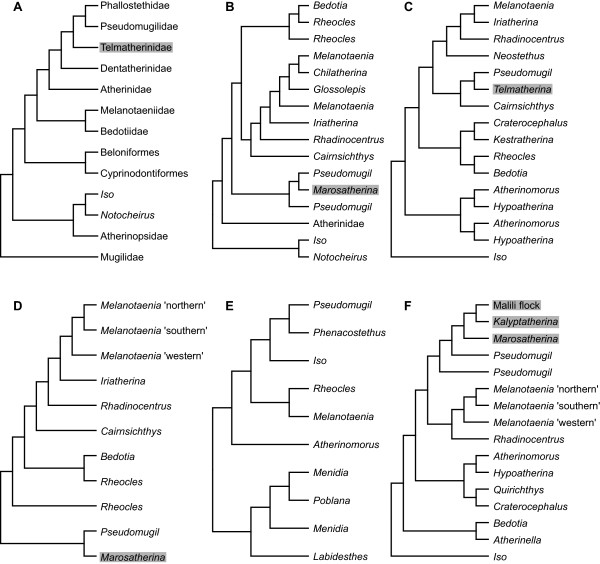
**Phylogenetic hypotheses showing the systematic position of Telmatherinidae and relatives among different studies. (A)**[39], **(B)**[22], **(C)**[32], **(D)**[24], **(E)**[40], **(F)** Present study. Telmatherinid representatives are highlighted in grey. See text for details.

The phylogenetic reconstructions show a well to highly supported sister group relationship between Melanotaeniidae and a clade comprising species of the genus *Pseudomugil* (Pseudomugilidae) and all representatives of the family Telmatherinidae (Figure 2 and node 6 in Figure 1). Inferred mean ages for the split (node 6) range from 23.6-75.9 My among the four molecular analyses performed in BEAST (Table 2). Basal relationships among atherinid, atherinopsid and bedotiid species are not well resolved in ML, BI and BEAST analyses, and estimated mean ages for these basal nodes are strikingly different and range between 31.3 and 103.1 My for the root height (node 1) and the first basal split (node 2: 28.7-93.5 My).

The Melanotaeniidae form a highly supported monophyletic group, including *Rhadinocentrus ornatus* from Queensland, and all species of the genera *Melanotaenia*, *Chilatherina* and *Glossolepis* analyzed. In this group, *R. ornatus* represents the sister taxon to three distinct and highly supported clades from New Guinea (and surrounding islands), and northern Australia (Figure 1). Interestingly, *Glossolepis dorityi* and *G. pseudoincisus* share one haplotype (Figure 1), which is most likely due to gene flow between the two species inhabiting the very same river system in the northern lowlands of New Guinea. Two *Melanotaenia* species from the Bird’s Head (*M. angfa*) and Batanta Island (*M. batanta*) form a monophyletic group, being sister to two separate clades comprising *Melanotaenia* (sub)species from southern New Guinea and northern Australia, and species of the genera *Chilatherina*, *Glossolepis* and *Melanotaenia* from northern New Guinea (cf. “western”, “southern”, and “northern” clade in [24]; Figure 1). This is largely congruent with findings of [24]. The inferred mean age estimates for the western–northern/southern split (node 8 in Figure 1) and the northern–southern split (node 9 in Figure 1) range from 12.7-40.8 and 10.9-34.5 My, respectively, depending on if node 9 was enforced in the respective analysis (see Table 2, analyses [B and D]).

Phylogenetic reconstructions (ML and BI) show a well to highly supported clade comprising members of the genus *Pseudomugil* (*P. signifer*, *P. reticulatus*, *P. furcatus*, and *P. pellucidus*; note that the Pseudomugilidae are paraphyletic; see also below), and the Telmatherindae. Within the Telmatherinidae, *Marosatherina ladigesi* from the Maros karst (southwest Sulawesi) is basal to the clade including *Kalyptatherina helodes* (Batanta and Misool) plus the sailfin silversides from the Malili Lakes system in Central Sulawesi, Indonesia (node 14; mean age range: 8.5-28.9 My). Estimated divergence times range from 18.1-57.8 My for the split of Telmatherinidae and the most recent Pseudomugilidae clade (node 11), and are quite similar for the first diversification events within each family (12.3-37.9 My for node 12 for the most recent *Pseudomugil* clade, and 12.9-42.9 My for node 13, respectively).

Three different genera belong to the Malili Lakes species flock, namely *Paratherina*, *Tominanga*, and *Telmatherina* (node 15 = sailfin silversides split: 5.2-17.4 My). The morphologically and nuclear distinct *Paratherina* (Lakes Mahalona and Towuti; node 24 = TMRCA, time to most recent common ancestor of *Paratherina* and *Telmatherina*: 3.0-10.2 My, node 27 = 'speciation’ , the first intra-clade diversification within *Paratherina*: 1.6-5.1 My) and *Tominanga* (Lake Mahalona; node 21 = TMRCA of *Tominanga* and *Telmatherina*: 1.2-3.6 My, node 23 = 'speciation’: 0.4-1.2 My) are both clearly supported as monophyletic. In contrast, species of the genus *Telmatherina* fall in three distinct mitochondrial clades. Node 25 (1.8-6.1 My) represents the MRCA of a clade (from now on we refer to MRCAs of particular clades when discussing nodes) is composed of the lake-dwelling *Telmatherina celebensis* from Lakes Mahalona and Towuti, and several populations of stream-dwelling *Telmatherina bonti*. Node 22 (0.4-1.2 My) comprises sharpfin specimens from Lake Matano, together with stream-dwellers; node 16 contains the remaining *Telmatherina* from Lake Matano, namely sharpfins (node 19), roundfins (node 18), and two stream *Telmatherina* from River Nuha (north of Matano) and River Tominanga (draining Lake Mahalona to Towuti). Taken together, these results are consistent with those reported by [17]. In the light of nuclear and morphological data (cf. [17]), the mitochondrial signatures provide evidence for substantial hybridization between lake- and stream-dwelling *Telmatherina*. This results in two haplotype clades, the “original” Matano haplotypes (node 19) being sister to the endemic roundfins (node 18), and the introduced haplotypes of (node 22) within Lake Matano’s sharpfins.

**Figure 3:**
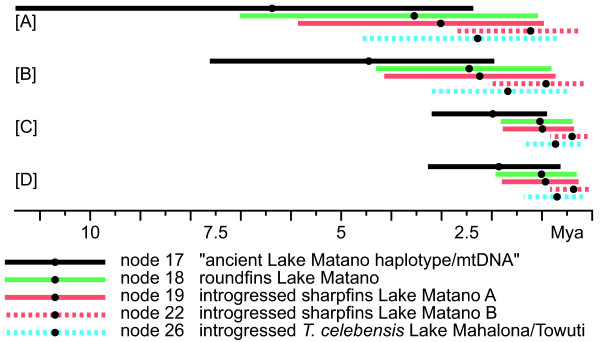
**Relaxed-clock divergence times distribution among analyses for the Lake Matano telmatherinid radiation.** Bars denote 95% credibility intervals, dots represent mean ages for MRCAs of the respective clades. See Methods and Table 2 for details.

The comparison of diversification and mitochondrial introgression events within the Lake Matano radiation suggests that the ancient clade of haplotypes, endemic to the lake (node 17), is approximately 1.9-6.4 My old. It comprises the clades of the lake’s two sub-radiations, namely the pelagic to benthopelagic roundfins (node 18: 1.0-3.7 My), and the “native” sharpfin haplotypes of the lake (node 19: 0.9-3.1 My). In contrast, the age of the introgressed haplotypes present in parts of the sharpfin individuals (node 22) is apparently more recent (0.4-1.2 My). A comparatively recent origin of mitochondrial introgression is also supported for the second case of massive hybridization of stream- and lake-haplotypes: node 26, representing the *Telmatherina celebensis* clade from Lakes Mahalona and Towuti, is estimated to only 0.7-2.4 My (see Figure 3 and Table 2 for details).

## Discussion

The endemic sailfin silversides radiation of the Malili Lakes serves as a model system in evolutionary ecology research (see [10,19,41] for reviews). The detailed and temporal reconstruction of the phylogenetic history of the *Telmatherinidae* is crucial for exploring the likely conditions underlying speciation processes, including the spatial origin and morphological traits of ancestral and introgressed populations. It remained, however, unclear if it is justified to consider *Marosatherina* from SW Sulawesi as the sister species to the lakes radiation, and if the age of the lakes radiation in fact falls into preliminary lake age estimations proposed by geologists and limnologists, which are however not yet fully reliable [41]. These preliminary seismic data suggest an age of at least 600,000-700,000 years for Lake Towuti [41], while the geological fault formation, in which Lake Matano is embedded, might be comparatively older possibly starting around 4 Mya [42]. An onset of the Malili Lakes system formation in the early Pleistocene c. 1–2 Mya is plausible (Robert Hall, pers. comm.).

### The closest relative to the sailfin silversides radiation

The phylogenetic reconstructions presented here are based on mitochondrial markers only, and hence do not allow inferences about cyto-nuclear discordances within the sailfin silversides species flock (cf. [17]). However, this mtDNA dataset allows inferring a first hypothesis for reconstructing phylogenetic relationships among disjunctly distributed telmatherinid species, whose mtDNA haplotypes most likely have preserved the vicariant phylogenetic signal.

Our analyses clearly suggest that mtDNA haplotypes of the Malili Lakes radiation are more closely related to *Kalyptatherina helodes,* the only telmatherinid species occurring on islands east of Sulawesi (Misool and Batanta), rather than to *Marosatherina ladigesi* from SW Sulawesi, a species previously considered the sister taxon to the Malili flock. Its inclusion into the Telmatherinidae is highly plausible in the light of morphological data [43], but its placement as the sister taxon to the lacustrine flocks appears surprising. However, reconstructions of the complex geological history of Sulawesi and adjacent islands may provide explanations for these findings (see section below).

The phylogenetic relationships of the families within Atheriniformes, and the composition of these families, remain partially controversial, most likely due to substantial differences in taxon sampling and the methods applied (Figure 2). Two important taxa could not be included in the present dataset, namely *Cairnsichthys rhombosomoides* (Queensland, Australia), and the rather widespread brackish water family Phallostethidae (priapumfishes; recorded from Sundaland, Luzon, Palawan and Southwest Sulawesi; [44,45]. *Cairnsichthys* is suggested to be basal to *Pseudomugil* and *Telmatherina* according to the molecular phylogeny by [24], while the phallostethids are placed as the sister to *Pseudomugil* according to [40]. Morphological studies by [39] suggested Melanotaeniidae as sister to Madagascar’s Bedotiidae, which are in turn most closely related to a clade composed of Telmatherinidae, Pseudomugilidae (blue eyes), and three other atheriniform families. The combined analysis of molecular and morphological data by [22] supported the sister group relationship of monophyletic Melanotaeniidae and Bedotiidae; a clade composed of *Marosatherina* (Telmatherinidae) and two *Pseudomugil* species (Pseudomugilidae) represent the sister clade to Melanotaeniidae and Bedotiidae in that study. A comprehensive study by [32] reported contrasting results, based on the combined analysis of one mitochondrial and nuclear marker. The single representatives of *Pseudomugil* and *Telmatherina* analyzed by [32] were also supported as most closely related, but nested within a clade composed of Melanotaeniidae and Phallostethidae. Most recently, [40] confirmed the inclusion of Phallostethidae within the atherinomorphs as distant sister to a monophyletic group of *Pseudomugil* species, based on 10 nuclear markers. Unmack et al. [24] found in a comprehensive multilocus molecular study (seven mtDNA markers and one nuclear marker) that Melanotaeniidae are the monophyletic sister group to Madagascar’s *Rheocles* and *Bedotia*; Melanotaeniidae were again supported as sister to a clade composed of *Marosatherina* and *Pseudomugil*. The results of the present study clearly support the close relationship between Telmatherinidae and Pseudomugilidae reported by previous workers. However, the Pseudomugilidae are also clearly paraphyletic, with *P. signifer* being basal to all remaining Pseudomugilidae and Telmatherinidae (Figure 1). This finding is consistent with the results of [22] (see also Figure 2), and highlights the need for a detailed study addressing the systematic position of several *Pseudomugil* species, especially that of *P. signifer*.

### Biogeographic implications

Divergence time estimates of the split of *Kalyptatherina* and the central Sulawesi Malili flock of 8.5-28.9 Mya renders a scenario of terrane-rafting the most plausible explanation for the present-day distribution pattern, given the region’s geological history. In contrast, and considering its present local distribution and estimated node ages, the ancestor of *Marosatherina* most likely colonized western Sulawesi by dispersal from the Sahul Shelf, the current distribution of *Pseudomugil* and Melanotaeniidae.

**Figure 4:**
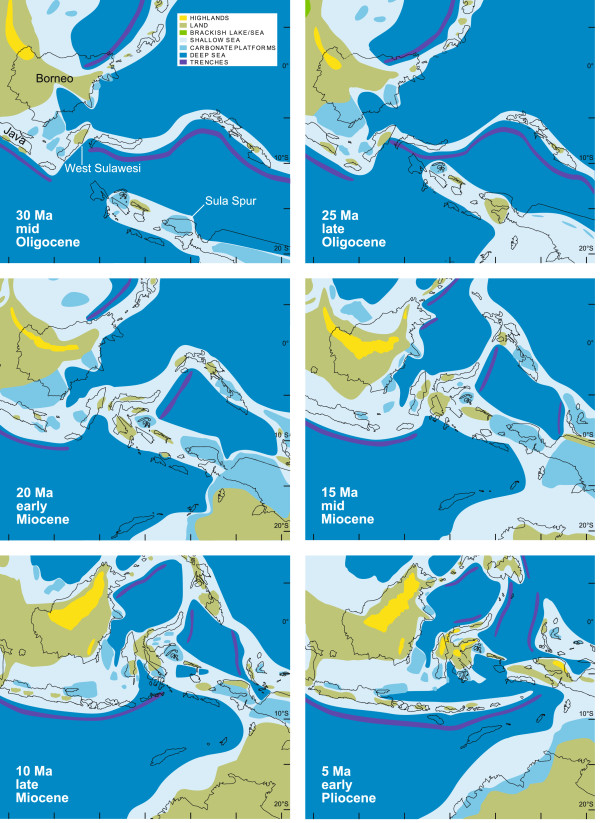
**Palaeogeographic maps of SE Asia, with particular focus on West Sulawesi and the Sula Spur.** Modified from [51], with permission (see text for details).

West Sulawesi (i.e., the West Sulawesi Plutono-Volcano Arc), was separated from the Asian margin when the Makassar Strait opened in the Eocene c. 45 Mya [46-48]. Extension and westward movements of the Sula Spur (a large promontory of the Australian margin) resulted in a collision with the North Sulawesi volcanic arc c. 20–23 Mya, and a final amalgamation of the Sula Spur (comprising East Sulawesi, Central Sulawesi and Banggai-Sula) with West Sulawesi in the Miocene c. 10–20 Mya (Figure 4; see [49,50] for more details on general terrane movements and distribution of land and sea in the Indo-Australian Archipelago, and particularly in Sulawesi). Geological separation and fusion represent constraints on vicariant processes involving the colonization of the island’s terrestrial and freshwater biota.

A scenario of 'terrane-rafting’ provides a plausible explanation for the sister group relationship between *Kalyptatherina*, endemic to the small islands off the Vogelkop Peninsula of New Guinea, and the Malili Lakes sailfin silversides. Geological elements formerly belonging to the Sula Spur were in proximity to the Australian margin – including old offshore islands like Batanta and Misool – before this promontory was extended, moved westwards, and finally collided with West Sulawesi (e.g., [37,47]). It appears most plausible that the population ancestral to the Malili sailfin silversides originates from the Sahul Shelf area, and was dispersed on such a 'terrane raft’ when the Sula Spur was extended and moved westwards until this fragment ('raft’) collided with West Sulawesi. However, given the temporal uncertainties in both the separation of the Sula Spur from the Sahul Shelf (c. 15 Mya; see also [50]) and divergence time estimates among the four analyses, it remains difficult to test this hypothesis. Mean ages and credibility intervals suggest that this scenario might be plausible for analyses [A] and [B] (15.0-43.5 My and 9.6-30.4 My; see Table 2), while the credibility intervals obtained from analyses [C] and [D] (5.5-13.1 and 3.8-13.5) would slightly post-date the estimated age of the Sula Spur separation. However, the lack of fossil remains requires denser sampling and the incorporation of multiple markers to explain the relationships of present-day geographically distant groups, which probably have been in vicinity in the past.

A marine dispersal explanation for the sister group relationship between *Marosatherina*, a monotypic genus endemic to the Maros karst in SW Sulawesi, and all the remaining sailfin silversides, appears most likely based on its current distribution and the divergence time estimates inferred (node 13: 12.9-42.9 My). West Sulawesi and the remaining geological parts of the island are of different origin (Sunda Shelf and Sahul Shelf), and amalgamated not until in the Miocene due to tectonic movements. It appears plausible to assume that the population ancestral to *Marosatherina* might have originated in the Sahul Shelf area and colonized present-day West Sulawesi by marine dispersal; this requires, however, the assumption that the dispersing proto-*Marosatherina* had a tolerance for marine conditions. Such a salt tolerance might indeed be a plesiomorphic character of sailfin silversides. The occurrence of *Kalyptatherina*, *Neostethus* (Phallostethidae, present with one species on Sulawesi, *Neostethus djajaorum*; [45]), and also some *Pseudomugil* species in brackish habitats, provides support for this assumption. Likewise, *Marosatherina* as well as *Telmatherina bonti* tolerate brackish waters under aquarium conditions (F.H., pers. obs.). However, none of the Sulawesi sailfin silversides has ever been reported from such habitats in nature, and it remains thus unclear, if the assumption that ancestral sailfin silversides were able to cross marine barriers, is in fact realistic. The endemism of all recent species of the family provides a substantial argument against profound abilities for marine dispersal. As an alternative, partly complementary scenario, the ancestral population of *Marosatherina* might have been widespread across both Sunda and Sahul Shelf areas, followed by extinction across large extents during periods of sea-level fluctuations. In that case, *Marosatherina* would represent a relict species as several areas of West Sulawesi remained above sea level or at least were covered only by shallow water during the island’s history according to palaeogeographic reconstructions [47,52]. However, this again assumes that the ancestral population might have been, at least to some degree, saltwater-tolerant.

In line with earlier workers [24], the present results support the monophyly of the rainbow fishes (Melanotaeniidae). As expected, its genera *Chilatherina* and *Glossolepis* are nested within *Melanotaenia*, and the three major geographic clades recovered correspond to the expected freshwater ecoregions of “southern”, “northern” and “western” New Guinea [24,53]. The island’s Central Highlands are the major barrier putatively separating the “northern” and “southern” clades, and provide an opportunity to estimate divergence rates also within the Telmatherinidae (see below).

### Divergence time estimation: The Malili Lakes radiation

Depending on the method applied, molecular clock approaches estimate the onset of the Malili Lakes radiation to 5.2-17.4 My, but the youngest estimate appears by far the most plausible, given the estimated timeframe for Sulawesi’s final amalgamation (see above). This implies ages of about 1.9 My for the initial split of the benthic sharpfins and the predominantly pelagic to benthopelagic roundfins, estimates of c. 1.0 My for divergence within these two lineages inside Lake Matano, and substantially less (0.4 My) for the lineage of haplotypes introgressed by stream populations into Matano’s sharpfins.

[21] provided a first age estimation for the divergence among the three mitochondrial haplotype clades present in Lake Matano’s *Telmatherina* radiation. This approach was based on a constant rate of evolution, and applied a genetic distance-age ratio of 1-2%/My. For the sailfin silversides endemic to Lake Matano, the deepest and according to geological data (see [42] and Robert Hall, pers. comm.) oldest lake of the system, these analyses suggested an age of 0.95-1.9 My separating roundfins (“Clade I” in [21]; see [19] for the identity of these clades) and sharpfins (“Clade II”). Haplotypes originating from streams and rivers, present in Lake Matano’s sharpfins due to introgressive hybridization [17], diverged from the lacustrine haplotypes (sharpfins + roundfins) in Roy et al.’s [21] analyses 1.85-3.7 My ago. Their ingroup dataset did however not include the other sailfin silversides species occurring outside of Lake Matano, from the remaining lakes, rivers, and streams of the Malili Lakes; it appears accordingly unclear if this framework is suited for providing reliable estimates for the relevant splits. In the present study, we combine geological and indirect calibration points, as well as recently suggested substitution rates, to estimate and carefully discuss the timing of the most relevant splits within the sailfin silversides radiation in a relaxed molecular clock framework.

### Analysis [A] – indirect calibration for the *Iso–Melanotaenia* split

We first used the split between *Iso hawaiiensis* and *Melanotaenia lacustris* as an indirect calibration point, obtained from a study of ricefishes (Adrianichthyidae) by [36], in analysis [A]. This approach provides an age of 17.4 My for the Malili Lakes radiation (node 15); Lake Matano’s roundfins are estimated to an age of 3.7 Mya (node 18) in that analysis (Table 2). Under analysis [A], the age of the Malili Lakes radiation (node 15) significantly predates the proposed age for the formation of present-day Sulawesi, namely the final amalgamation of the North and West arms of Sulawesi with the Sula Spur (c. 10–20 Mya; [47,50], as well as the geological evidence for the age of the Lake Matano.

Some technical issues might account for the observed inconsistencies between the proposed ages of these nodes, and the geological reconstruction of Sulawesi’s history and its ancient lakes. Dating based on indirect calibration points presupposes that adequate calibration points were used in the source analysis. The 21 fossil and six biogeographic calibration points used by [36] are remarkable, but all fall outside the atherinomorphs. These priors, plus possible saturation effects, may have led to the bias of an overestimation of splits within the atherinomorphs. A recently published, and more comprehensive dated phylogeny by [40], might justify this inference. There, the atherinomorphs are estimated to have originated c. 80 Mya, while the genera *Iso* and *Melanotaenia* are more recent compared to the split in Setiamarga et al.’s study ( [36]: 93–113 My vs. [40]: c. 50 My); however, some basal nodes remain unresolved in [40], and thus both genera do not form a sister group, as in [36]. Thus, we conclude that the inferred node ages from analysis [A] very likely substantially overestimate the age of the Malili Lakes radiation, as well as that of the intralacustrine speciation and hybridization processes.

### Analysis [B] and [C] – indirect calibration for *Melanotaenia* clades and rate of 1%/My

Estimated divergence times for the New Guinean northern and southern *Melanotaenia* clades obtained from Unmack et al.’s [24] study have been used as indirect calibration points in analysis [B]. In addition, we used the substitution rate of 1%/My suggested by [24] in their rainbowfish dataset (analysis [C]). Interestingly, analysis [B] shows comparable ages for node 8 and node 9 compared to [24], however, the resulting ucld.mean (uncorrelated lognormal relaxed clock mean) is 0.47%/My for this analysis. Accordingly, age estimates inferred from analysis [B] clearly predate the hypothesized starting point of the New Guinean Highlands uplift (node 9: 25.9 My). However, the MRCA of the *Melanotaenia* clade is 39.5 My, and is therefore quite similar to the divergence time estimates of the single *Melanotaenia* lineage in [40].

Likewise, divergence times differ remarkably between the few comparable nodes of analysis [C] and Unmack et al.’s [24] study (see Table 2). The considerable mismatch between analysis [C] and the study by [24] might be related to the different prior conditions in the respective analyses. With respect to the target question, the taxon sampling available is more complete in the present study. Differences in the resulting estimates may also be due to the genetic markers applied: substitution rates vary across genetic markers and among taxa, and might therefore cause deviating node ages though using the same prior substitution rate.

### Analysis [D] – geological calibration using the Central Highlands formation

In a final analysis, we used age estimations for the Central Highlands in New Guinea, and the clades of rainbow fish separated by this major barrier, for estimating the age of the relevant nodes in the sailfin silversides phylogeny (analysis [D]). The Central Highlands in New Guinea are a massive mountain range, spanning almost the entire island with a maximum altitude of c. 5,000 m above sea level. The continuous formation of the Central Highlands since the late Miocene (c. 10–14 Mya; [37]) clearly resulted in the interruption of former watersheds, and led to the present-day regions of freshwater biodiversity. This explains the presence of two well-resolved clades of rainbow fishes north and south of this mountain range ( [24,54], this study); the ancestral populations became most likely continuously separated. However, given the nature of geological events, which should in most cases be considered rather as continuous processes than as distinct, precise events, it appears problematic to reconstruct the exact timeframe in which the proposed ancestral melanotaeniid population in New Guinea was initially separated, giving raise to the two distinct clades observed to date.

Compared to the indirect calibration approach, the prior age of node 9 is substantially younger in analysis [D] (mean age: 10.9 My) than the posterior age inferred from analysis [A] (mean age: 34.5 My). This results in a likewise substantially more recent age of both, the onset of the Malili Lakes radiation, and that of its radiating clades (see Table 2 and Figure 3). Accordingly, node ages derived from analysis [D] appear more plausible than those from analyses [A] and [B]. However, the root height representing the age of *Iso* considerably underestimates the divergence time inferred from [36] by about 70 My, while in contrast, *Iso* is estimated to be c. 15 My younger in analysis [D] compared to the study by [40]. This underestimation may not only be due to conflicting topologies, the position of *Iso* within the atherinomorphs (see Discussion above), and the comparatively recent calibration point used (node 9), but might be also correlated with saturation effects in basal nodes by solely using mitochondrial markers.

*Telmatherina* of Lake Matano’s endemic sharpfin radiation carry either mitochondrial haplotypes closely related to those of the lakes’ roundfins, or those introgressed by riverine populations [17-19]. The age of the haplotypes introgressed into the Matano flock (mainly node 22) is comparatively young, estimated in analysis [D] to less than 400,000 years. In contrast, age estimates for the “native” Matano sharpfin haplotypes (node 19: 0.9 My), its sister – the roundfins (node 18: 1.0 My) –, and the clade of *T. celebensis* from the lower lakes of the system (node 26: 0.7 My) are comparatively older, and appear largely congruent to the general age estimates for the Malili Lakes (see above). It appears therefore very likely that the mitochondrial introgression observed has occurred rather recently, in comparison to the age of the lakes’ native haplotypes (see Figure 3). Shared haplotypes in highly distinct lake- and stream-dwelling *Telmatherina* are also observed in Lakes Towuti’s and Mahalona’s *T. celebensis*, and several populations of *T. bonti* (see also Figure 1 and supplementary figure one in [17], incorporating more stream populations). Node 26, comprising these lacustrine and riverine populations, is estimated to c. 700,000 years ago in analysis [D], a time that coincides with the proposed age for Lake Towuti [41].

Similarly to the Lake Matano *Telmatherina* radiation, *Paratherina* represents a small, monophyletic radiation, occurring in off- and inshore waters of Lakes Mahalona and Towuti. Analysis [D] suggests that the first diversification event within *Paratherina* (node 27) has occurred c. 1.6 Mya. It appears reasonable to assume that the onset of diversification may have taken place in the older Lake Mahalona, from where the putatively younger Lake Towuti has been colonized. The *Paratherina* populations of both lakes are likely still connected via Tominanga River, as suggested by the recent microsatellite study of [55]. Further support for possible riverine dispersal of *Paratherina* comes from the historical presence of species shared with Lake Towuti in the small hill-lake Lontoa (or Wawontoa; see [56]), connected to the large lake by rivers. This lake has however undergone substantial degradation, and the presence of *Paratherina* could not be confirmed during recent surveys (F.H., pers. obs.).

## Conclusions

Divergence times inferred for the Malili Lakes radiation clearly predate both the final formation of Sulawesi and any suggested age estimates for the Malili Lakes for the majority of analyses performed. Hence, node ages derived from analysis [D], based on geological calibration by the New Guinean highland barrier, seem most plausible to us. This means that divergence times obtained from other sources, i.e., [24,36], possibly overestimate telmatherinid and melanotaeniid clade ages, again highlighting the issues related with molecular clock analyses (see Discussion above).

Some concluding remarks can be made on speciation and hybridization processes in the sailfin silversides radiation, based on the – in our view – most plausible analysis [D]. Based on the present topology, riverine *Telmatherina bonti* populations not only cluster within the lacustrine clades, indicating hybridization events; some of these riverine lineages also appear basal to some lacustrine populations. In line with similar results from an earlier phylogenetic study [17], this clearly indicates that the Malili Lakes were colonized by riverine populations, which appears highly plausible, and meets patterns observed also in invertebrate radiations, like e.g., the pachychilid snails [57].

The present study indicates that the Sulawesi telmatherinids might have originated c. 3–5 Mya, a period when present-day Sulawesi was being formed through a series of tectonic events such as e.g., mountain uplifts in West Sulawesi and the Matano fault formation [42,52]. The Matano fault gave rise to the rift lake Matano, and probably also initiated the formation of the remaining lakes of the Malili Lakes system. Preliminary geological reconstructions suggest that the Malili Lakes are generally 1–2 My old, while preliminary seismic data support with 600,000-700,000 years a younger age for Lake Towuti. This geological and seismic evidence is in line with the present phylogenetic reconstruction, which shows comparatively recent diversification and hybridization events within *Telmatherina celebensis* and their riverine relatives, compared to diversification and introgression in Lake Matano (node 26; see Figures 1 and 3). Age estimates suggest diversification along a benthic-pelagic axis, into sharpfins and roundfins, c. 1.9 Mya after Lake Matano was colonized by stream populations, followed by a rapid radiation in both of these clades in the last 1 My. Secondary hybridization did probably not affect initial divergence within Lake Matano’s sharpfin radiation, as the age of the introgressed haplotypes clearly postdates the initial diversification by about 600,000 years.

## Availability of supporting data

BEAST MCC tree files of analyses [A]-[D] are available in the Dryad repository, doi:10.5061/dryad.8dh7r at http://datadryad.org[58].

## Competing interests

The authors declare that they have no competing interests.

## Authors’ contributions

UKS and FH conceived and designed the study. FH and RKH conducted fieldwork in Sulawesi; RKH provided access to *Kalyptatherina* samples. IS performed laboratory work, BS conducted the analyses. BS, FH and UKS wrote the manuscript. All authors read and approved the final manuscript.

## Supplementary Material

Additional file 1: Table S1 List of studied specimens including distribution information and accession numbers [59]. Click here for file
