# Supplementary material for: Age estimates for an adaptive lake fish radiation, its mitochondrial introgression, and an unexpected sister group: Sailfin silversides of the Malili Lakes system in Sulawesi
Source: BMC Evol Biol. 2014 May 3;14:94. doi: 10.1186/1471-2148-14-94 (PMC4029975; doi:10.1186/1471-2148-14-94)
Supplement: Additional file 1: Table S1 — List of studied specimens including distribution information and accession numbers [59]. [file 1471-2148-14-94-S1.doc]

**Additional file 1: Table S1. List of studied specimens including distribution information and accession numbers**

| **Family** | **Species** | **Voucher no. or GenBank no.^1^** | **Locality/distribution (AS = Aquarium stock)** | **Accession no.**  **ND2** | **Accession no.**  **12S-16S** |
| --- | --- | --- | --- | --- | --- |
| Notocheiridae | *Iso rhotophilus* | ZSM (RS10)* | Australia | KJ667882 | KJ667787 |
| Atherinidae | *Atherinomorus endrachtensis* | ZSM (RS3)* | Indo-Pacific | KJ667867 | KJ667772 |
|  | *Atherinomorus lacunosus* | ZSM (RS5)* | Japan | KJ667868 | KJ667773 |
|  | *Craterocephalus honoriae* | ZSM (RS6)* | Australia | KJ667877 | KJ667782 |
|  | *Hypoatherina tsurugae* | AP004420 | Gregory River, Queensland, Australia | [59] | [59] |
|  | *Quirichthys* sp*.* | ZSM 34172-1 | Gregory River, Queensland, Australia | KJ667922 | KJ667824 |
|  | *Quirichthys* sp*.* | ZSM 34172-3 | Gregory River, Queensland, Australia | KJ667923 | KJ667825 |
| Atherinopsidae | *Atherinella panamensis* | ZSM (RS4)* | Panama (Eastern Pacific) | KJ667866 | KJ667771 |
| Bedotiidae | *Bedotia* sp. | ZSM 34169 | Ankavia, Madagascar (AS) | KJ667870 | KJ667775 |
|  | *Bedotia* sp. | ZSM 34168 | Lazana, Madagascar (AS) | KJ667869 | KJ667774 |
| Melanotaeniidae | *Chilatherina bleheri* | ZSM 34140 | Papua New Guinea (AS) | KJ667872 | KJ667777 |
|  | *Chilatherina campsi* | ZSM 34177 | Papua New Guinea (AS) | KJ667873 | KJ667778 |
|  | *Chilatherina fasciata* | ZSM 34137 | Clearwater Creek, Papua New Guinea (AS) | KJ667874 | KJ667779 |
|  | *Chilatherina fasciata* | ZSM 34142 | Lake Wanam, Papua New Guinea (AS) | KJ667876 | KJ667781 |
|  | *Chilatherina fasciata* | ZSM 34141 | Lake Sentani, West Papua, Indonesia (AS) | KJ667875 | KJ667780 |
|  | *Chilatherina* sp. | ZSM 34143 | Mamberamo River, West Papua, Indonesia (AS) | KJ667871 | KJ667776 |
|  | *Glossolepis dorityi* | ZSM (IS50)* | Lake Nenggwambu, West Papua, Indonesia (AS) | KJ667878 | KJ667783 |
|  | *Glossolepis maculosus* | ZSM 34147 | Aquarium stock | KJ667879 | KJ667784 |
|  | *Glossolepis pseudoincisus* | ZSM (IS52)* | Tami River System, Lake Yaniruk, West Papua, Indonesia (AS) | KJ667880 | KJ667785 |
|  | *Glossolepis romuensis* | ZSM (IS51)* | Ramu River System, Papua New Guinea (AS) | KJ667881 | KJ667786 |
|  | *Melanotaenia affinis* "Lae" | ZSM 34138 | Aquarium stock | KJ667888 | KJ667789 |
|  | *Melanotaenia affinis* "Pagwi" | ZSM 34146 | Aquarium stock | KJ667889 | KJ667790 |
|  | *Melanotaenia angfa* | ZSM 34148 | Aquarium stock | KJ667890 | KJ667791 |
|  | *Melanotaenia angfa* | ZSM 34149 | Aquarium stock | KJ667891 | KJ667792 |
|  | *Melanotaenia australis* | ZSM (IS57)* | Northern Australia (AS) | KJ667892 | KJ667793 |
|  | *Melanotaenia batanta* | ZSM 34535 | Batanta Island, West Papua, Indonesia (AS) | KJ667893 | KJ667794 |
|  | *Melanotaenia boesemani* | ZSM 34153 | Lake Aitinyo, West Papua, Indonesia (AS) | – | KJ667795 |
|  | *Melanotaenia cf. eachamensis* | ZSM 34158 | upper Barron River, Queensland, Australia (AS) | KJ667894 | KJ667796 |
|  | *Melanotaenia douboulayi* | ZSM (IS54)* | Eastern Australia (AS) | KJ667895 | KJ667797 |
|  | *Melanotaenia goldiei* "Tapini" | ZSM 34174 | Aquarium stock | KJ667896 | KJ667798 |
|  | *Melanotaenia herbertaxelrodi* | ZSM (IS53)* | Lake Tebera, Papua New Guinea (AS) | KJ667897 | KJ667799 |
|  | *Melanotaenia lacustris* | AP004419 | Lake Kutubu, Papua New Guinea (AS) | – | [59] |
|  | *Melanotaenia lacustris* | ZSM (IS58)* | Lake Kutubu, Papua New Guinea (AS) | KJ667898 | KJ667800 |
|  | *Melanotaenia macchullochi* | ZSM (IS61)* | Eastern Australia (AS) | KJ667899 | KJ667801 |
|  | *Melanotaenia papuae* | ZSM 34151 | Aquarium stock | KJ667900 | KJ667802 |
|  | *Melanotaenia praecox* | ZSM (IS55)* | Mamberamo River System, West Papua, Indonesia (AS) | KJ667901 | KJ667803 |
|  | *Melanotaenia sexlineata* "Kiunga" | ZSM 34155 | Aquarium stock | KJ667903 | KJ667805 |
|  | *Melanotaenia sexlineata* "Tabubil" | ZSM 34154 | Aquarium stock | KJ667902 | KJ667804 |
|  | *Melanotaenia splendida inornata* | ZSM 34167 | Adelaide River, Northern Territory, Australia (AS) | KJ667908 | KJ667810 |
|  | *Melanotaenia splendida inornata* | ZSM 34161 | Blyth River, Northern Territory, Australia (AS) | KJ667904 | KJ667806 |
|  | *Melanotaenia splendida inornata* | ZSM 34165 | Flat Rock Creek, New South Wales, Australia (AS) | KJ667906 | KJ667808 |
|  | *Melanotaenia splendida inornata* | ZSM 34163 | Goyder Creek, Northern Territory, Australia (AS) | KJ667905 | KJ667807 |
|  | *Melanotaenia splendida inornata* | ZSM 34166 | Mann River, New South Wales, Australia (AS) | KJ667907 | KJ667809 |
|  | *Melanotaenia splendida splendida* | ZSM 34160 | Deepwater Creek, Queenslands, Australia (AS) | KJ667909 | KJ667811 |
|  | *Melanotaenia trifasciata* "Hapgood River" | ZSM 34180 | Aquarium stock | KJ667911 | KJ667813 |
|  | *Melanotaenia trifasciata* | ZSM 34182 | Pappan Creek, Queensland, Australia (AS) | KJ667912 | KJ667814 |
|  | *Melanotaenia trifasciata* | ZSM 34157 | Wonga Creek, Norther Territory, Australia (AS) | KJ667910 | KJ667812 |
|  | *Rhadinocentrus ornatus* | ZSM 34156 | Coolum Creek, Queensland, Australia (AS) | KJ667924 | KJ667826 |
| Pseudomugilidae | *Pseudomugil furcatus* | ZSM (IS68)* | Aquarium stock | KJ667918 | KJ667820 |
|  | *Pseudomugil pellucidus* | ZSM (IS63)* | Aquarium stock | KJ667919 | KJ667821 |
|  | *Pseudomugil reticulatus* | ZSM 34170 | Aquarium stock | KJ667920 | KJ667822 |
|  | *Pseudomugil signifer* | ZSM (IS66)* | Aquarium stock | KJ667921 | KJ667823 |
| Telmatherinidae | *Kalyptatherina helodes* | MZB 17152-1 | Batanta, west of New Guinea | KJ667883 | – |
|  | *Kalyptatherina helodes* | MZB 17152-2 | Batanta, west of New Guinea | KJ667884 | – |
|  | *Kalyptatherina helodes* | MZB 22095-1 | Misool, west of New Guinea | KJ667885 | – |
|  | *Kalyptatherina helodes* | MZB 22095-2 | Misool, west of New Guinea | KJ667886 | – |
|  | *Marosatherina ladigesi* | ZSM 34735 | Maros karst, SW Sulawesi, Indonesia | KJ667887 | KJ667788 |
|  | *Paratherina* cf*. labiosa* | ZSM 33054 | Lake Mahalona, Sulawesi, Indonesia | KJ667913 | KJ667815 |
|  | *Paratherina* cf*. labiosa* | ZSM 33055 | Lake Mahalona, Sulawesi, Indonesia | KJ667914 | KJ667816 |
|  | *Paratherina* cf*. striata* | ZSM 33057 | Lake Mahalona, Sulawesi, Indonesia | KJ667915 | KJ667817 |
|  | *Paratherina* cf*. striata* | ZSM 33061 | Lake Towuti, Sulawesi, Indonesia | KJ667916 | KJ667818 |
|  | *Paratherina striata* | ZSM 33062 | Lake Mahalona, Sulawesi, Indonesia | KJ667917 | KJ667819 |
|  | *Telmatherina sarasinorum* | ZSM 33115 | Lake Matano, Sulawesi, Indonesia | KJ667957 | KJ667859 |
|  | *Telmatherina sarasinorum* | ZSM 33116 | Lake Matano, Sulawesi, Indonesia | KJ667954 | KJ667856 |
|  | *Telmatherina sarasinorum* | ZSM 33117 | Lake Matano, Sulawesi, Indonesia | KJ667955 | KJ667857 |
|  | *Telmatherina sarasinorum* | ZSM 33127 | Lake Matano, Sulawesi, Indonesia | KJ667956 | KJ667858 |
|  | *Telmatherina abendanoni* | ZSM 33118 | Lake Matano, Sulawesi, Indonesia | KJ667926 | KJ667828 |
|  | *Telmatherina abendanoni* | ZSM (T26)* | Lake Matano, Sulawesi, Indonesia | KJ667925 | KJ667827 |
|  | *Telmatherina antoniae* | ZSM 33089 | Lake Matano, Sulawesi, Indonesia | KJ667927 | KJ667829 |
|  | *Telmatherina antoniae* | ZSM 33090 | Lake Matano, Sulawesi, Indonesia | KJ667928 | KJ667830 |
|  | *Telmatherina antoniae* | ZSM 33092 | Lake Matano, Sulawesi, Indonesia | KJ667929 | KJ667831 |
|  | *Telmatherina antoniae* | ZSM 33093 | Lake Matano, Sulawesi, Indonesia | KJ667930 | KJ667832 |
|  | *Telmatherina antoniae* | ZSM 33100 | Lake Matano, Sulawesi, Indonesia | KJ667931 | KJ667833 |
|  | *Telmatherina bonti* | ZSM 33067 | Stream close by Malili, Central Sulawesi, Indonesia | KJ667934 | KJ667836 |
|  | *Telmatherina bonti* | ZSM 33068 | Stream close by Malili, Central Sulawesi, Indonesia | KJ667935 | KJ667837 |
|  | *Telmatherina bonti* | ZSM 33070 | Tominanga River (connecting Mahalona-Towuti), Sulawesi, Indonesia | KJ667936 | KJ667838 |
|  | *Telmatherina bonti* | ZSM 33128 | Nuha, Lake Matano, Sulawesi, Indonesia | KJ667937 | KJ667839 |
|  | *Telmatherina bonti* | ZSM 33129 | Nuha, Lake Matano, Sulawesi, Indonesia | KJ667938 | KJ667840 |
|  | *Telmatherina bonti* | ZSM 33160 | Tominanga River (connecting Mahalona-Towuti), Sulawesi, Indonesia | KJ667939 | KJ667841 |
|  | *Telmatherina bonti* | ZSM (T85)* | Lake Towuti, Sulawesi, Indonesia | KJ667932 | KJ667834 |
|  | *Telmatherina bonti* | ZSM (T86)* | Lake Towuti, Sulawesi, Indonesia | KJ667933 | KJ667835 |
|  | *Telmatherina celebensis* | ZSM 33071 | Lake Mahalona, Sulawesi, Indonesia | KJ667940 | KJ667842 |
|  | *Telmatherina celebensis* | ZSM 33072 | Lake Towuti, Sulawesi, Indonesia | KJ667941 | KJ667843 |
|  | *Telmatherina celebensis* | ZSM 33078 | Lake Mahalona, Sulawesi, Indonesia | KJ667942 | KJ667844 |
|  | *Telmatherina celebensis* | ZSM 33079 | Lake Towuti, Sulawesi, Indonesia | KJ667943 | KJ667845 |
|  | *Telmatherina* cf*. obscura* | ZSM 33126 | Lake Matano, Sulawesi, Indonesia | KJ667944 | KJ667846 |
|  | *Telmatherina* cf*. wahjui* | ZSM (T53)* | Petea River (connecting Matano-Mahalona), Sulawesi, Indonesia | KJ667945 | KJ667847 |
|  | *Telmatherina* cf*. wahjui* | ZSM (T54)* | Petea River (connecting Matano-Mahalona), Sulawesi, Indonesia | KJ667946 | KJ667848 |
|  | *Telmatherina opudi* | ZSM 33120 | Lake Matano, Sulawesi, Indonesia | KJ667947 | KJ667849 |
|  | *Telmatherina opudi* | ZSM 33153 | Lake Matano, Sulawesi, Indonesia | KJ667948 | KJ667850 |
|  | *Telmatherina opudi* | ZSM 33154 | Lake Matano, Sulawesi, Indonesia | KJ667949 | KJ667851 |
|  | *Telmatherina opudi* | ZSM 33157 | Lake Matano, Sulawesi, Indonesia | KJ667950 | KJ667852 |
|  | *Telmatherina prognatha* | ZSM 33097 | Lake Matano, Sulawesi, Indonesia | KJ667951 | KJ667853 |
|  | *Telmatherina prognatha* | ZSM 33106 | Lake Matano, Sulawesi, Indonesia | KJ667952 | KJ667854 |
|  | *Telmatherina prognatha* | ZSM 33110 | Lake Matano, Sulawesi, Indonesia | KJ667953 | KJ667855 |
|  | *Telmatherina* sp. | ZSM 33121 | Lake Matano, Sulawesi, Indonesia | KJ667958 | KJ667860 |
|  | *Telmatherina* sp. | ZSM 33122 | Lake Matano, Sulawesi, Indonesia | KJ667959 | KJ667861 |
|  | *Telmatherina wahjui* | ZSM 33151 | Lake Matano, Sulawesi, Indonesia | KJ667961 | KJ667863 |
|  | *Telmatherina wahjui* | ZSM (T33)* | Lake Matano, Sulawesi, Indonesia | KJ667960 | KJ667862 |
|  | *Tominanga* sp*.* | ZSM 33133 | Lake Mahalona, Sulawesi, Indonesia | KJ667962 | KJ667864 |
|  | *Tominanga* sp*.* | ZSM 33134 | Lake Mahalona, Sulawesi, Indonesia | KJ667963 | KJ667865 |

^1^ MZB = Museum Zoologicum Bogoriense, Bogor; ZSM = Zoologische Staatssammlung München (Bavarian State Collection of Zoology, Munich); * = no voucher material available.
